# Curiosity in younger and older adults: the relationship between information value and memory

**DOI:** 10.3389/fcogn.2026.1715793

**Published:** 2026-03-13

**Authors:** Michelle E. Hirsch, William Fisher, Andrée-Ann Cyr

**Affiliations:** 1 Department of Psychology, York University, Toronto, ON, Canada; 2 Department of Psychology, York University – Glendon Campus, Toronto, ON, Canada

**Keywords:** aging, curiosity, information prediction error, motivation, reward

## Abstract

**Introduction:**

Curiosity is a fundamental drive experienced throughout the lifespan. Beyond its health benefits, curiosity is associated with enhanced memory: Greater curiosity about information predicts improved recall in both younger and older adults. Recent work indicates that not only curiosity but also satisfaction with information and information prediction errors (IPEs)—the discrepancy between curiosity and satisfaction—can influence memory. However, less is known about how aging influences these affective variables.

**Methods:**

In this study, younger and older adults viewed trivia questions and rated their curiosity to learn the answers and their confidence in their knowledge of the answer. After the answer was shown, participants rated how satisfying they found it.

**Results:**

No age differences were observed: Across both age groups, confidence, satisfaction, and IPEs similarly predicted recall memory. In contrast, curiosity did not predict better recall memory in either age group, suggesting that satisfaction with information and IPEs play a more central role in learning trivia answers.

**Discussion:**

Overall, these findings show that pre- and post-information evaluations work cooperatively and independently to support memory across the lifespan.

## Introduction

Epistemic curiosity, i.e., the drive to acquire new knowledge, is a ubiquitous motivation involved in nearly all aspects of human and non-human animal learning (Kidd and Hayden, 2015; Modirshanechi et al., 2023). Curiosity is often associated with childhood, a period of front-loaded exploration aimed at building the foundational knowledge needed for adulthood and shaping personal and educational development. However, we know that curiosity continues to be expressed throughout the lifespan (Giambra et al., 1992) and is accompanied by a host of health benefits (Consedine and Moskowitz, 2007; Daffner et al., 2006; Lydon-Staley et al., 2020; Swan and Carmelli, 1996). Indeed, higher curiosity is tied to enhanced well-being in older age (Sakaki et al., 2018) and its loss can be an early sign of Alzheimer's disease (Daffner et al., 1992). As we age, continued interest in the novel aspects of our environments and engagement with cognitively stimulating activities may create a buffer against cognitive decline and promote longevity.

Potential age-related changes to epistemic curiosity remain unclear, however. Scores on self-report measures of trait curiosity (i.e., one's characteristic levels of curiosity that are stable across time) are lower among older relative to younger adults (Chu and Fung, 2022; Chu et al., 2020; Robinson et al., 2017; Whatley et al., 2025; Yagi et al., 2023), consistent with age-related decreases in openness to experience (Specht et al., 2011; Terracciano et al., 2005; Wortman et al., 2012; Zimprich et al., 2009) and sensitivity to novelty (Czigler et al., 2006; Shan et al., 2023). By contrast, state curiosity, one's moment-to-moment experiences of curiosity, appears to be relatively spared. For instance, studies show that being in a state of higher relative to lower curiosity leads to better learning and memory for both younger and older adults (Chu and Fung, 2022; Galli et al., 2018; McGillivray et al., 2015; Padulo et al., 2022; Swirsky et al., 2021; Swirsky and Spaniol, 2024; Whatley et al., 2025).

The most commonly used method to measure curiosity's effect on memory is the trivia paradigm, whereby participants are shown a series of general knowledge trivia questions one at a time (e.g., *What is the first country to give women the right to vote?*), and asked to rate how curious they are to know the answers which are then presented (e.g., *New Zealand*). During the test phase, participants are shown the trivia questions again and asked to recall the correct answer. Among younger adults, higher initial curiosity for trivia questions predicts better memory for answers after delay (Fastrich et al., 2018; Gruber and Ranganath, 2019; Kang et al., 2009). These findings have been replicated in older participants: Curiosity-driven learning is equivalent or better among older adults (Chu and Fung, 2022; Galli et al., 2018; McGillivray et al., 2015; Swirsky et al., 2021; Swirsky and Spaniol, 2024). This equivalence may be surprising given that the neural mechanisms that underpin curiosity-based memory enhancement overlap with those involved in extrinsic reward-processing, namely dopamine rich brain regions and the hippocampus (Gruber et al., 2014; Gruber and Ranganath, 2019). Aging is associated with a decline in both dopaminergic function (Bäckman et al., 2006; Eppinger et al., 2012) and episodic memory (see Park et al., 2002) yet older adults still benefit from the effects of epistemic curiosity. One possibility is that epistemic emotions such as curiosity and interest are positive states which may help guide older adults' memory processes toward information that is deemed interesting. Indeed, it is well established that age differences in memory can be attenuated when motivational factors such as personal relevance (Hess et al., 2009; Hess, 2014; Rahhal et al., 2002), or emotional valence (see Mather and Carstensen, 2005) are equated, and curiosity may be another such modifier.

While curiosity may direct attention toward information, how one feels once curiosity is resolved may also be an important predictor of memory. For example, a person may be curious about the answer to a trivia question but ultimately disappointed in the answer. Alternatively, an answer could be considered surprising or interesting, leading to greater satisfaction. McGillivray et al. (2015) asked younger and older participants to rate their curiosity for trivia answers as well as their post-answer interest. They found that post-answer interest—but not initial curiosity—predicted later memory for answers among both age groups. Moreover, the supportive effects of post-answer interest increased over a delay for older but not younger adults, indicating that this may be an especially important affective variable for older adults. Fandakova and Gruber (2021) conducted a similar study among children and adolescents and found that older learners were significantly more likely to remember answers that received higher as compared to lower post-answer interest ratings. These studies suggest that reflecting with interest on information that evoked curiosity is beneficial for learning, especially as we age.

Moreover, studies have applied a reinforcement learning framework to investigate how pre- and post-information affect interact to support memory performance. Marvin and Shohamy (2016) examined whether the discrepancy between initial curiosity (the expected reward value) and post-answer interest (the actual reward value) drives learning. They calculated so-called information prediction errors (IPE) which essentially represent the extent to which individuals are “surprised” by the information. If the obtained information is unexpected, information prediction errors are positive (i.e., individuals are surprised) and the value of information increases. This is consistent with previous research on the hypercorrection effect which shows that people better remember information that contradicts their expectations (e.g., learning that the capital of Australia is Canberra and not Sydney as one strongly expected; see Metcalfe, 2017). By contrast, if the new information is not more than what one expected, one may be disappointed and the value of information may be lowered (e.g., learning that Sydney is Australia's most populous city as expected). The idea that curiosity is partly driven by predictions is central to Loewenstein (1994) information gap theory which argued that when a knowledge gap is made salient, the degree to which people are motivated to seek information (i.e., to resolve the knowledge gap) depends on the “expected” value of the information. Marvin and Shohamy found that trivia answers that elicited greater post-answer satisfaction than initial curiosity (i.e., positive IPEs) predicted better memory relative to negative IPEs (see also Fandakova and Gruber, 2021; Fastrich et al., 2018; Ligneul et al., 2018).

However, no study has investigated whether IPEs modulate memory to the same extent among older adults. A recent study asked younger and older participants to rate their initial curiosity for trivia answers as well as their level of surprise (Sobczak et al., 2025): Although increased curiosity promoted better recall memory in younger and older adults, the effect of surprise on memory was stronger in younger participants. Prediction errors are strongly linked to the dopamine system, i.e., activity in the ventral striatum, midbrain and prefrontal cortex, areas which are known to undergo age-related deterioration (Bäckman et al., 2006; Eppinger et al., 2013). Moreover, studies using probabilistic learning paradigms have found a reduction in the neural representation of prediction errors among older adults (Chowdhury et al., 2013; Eppinger et al., 2013; Samanez-Larkin et al., 2014). As such, it is possible that IPEs may not drive learning to the same extent among older relative to younger adults. Accordingly, the goal of the present study was to investigate the contributions of pre-information curiosity, post-information satisfaction, and information prediction errors to episodic memory among healthy younger and older adults. Here, we employed a trivia paradigm whereby participants were asked to rate their curiosity to know the trivia answers on a rating scale. Given evidence that curiosity and satisfaction predict memory across the lifespan, we expected similar effects of pre-answer curiosity, and post-answer satisfaction on recall memory in older and younger adults. We expected IPE would predict memory performance in younger adults, but that it would be reduced among older adults. Confidence ratings were also assessed as they could indicate strength of knowledge in the topic area.

## Method

### Participants

An a priori power analysis conducted in G^*^Power (Faul et al., 2007) indicated that n = 119 participants would be required to detect a medium effect (f^2^ = 0.15) with α = 0.05 and 95% power in a multiple regression model with three predictors. Our final sample (*n* = 160) exceeded this requirement, providing greater precision and sensitivity relative to the planned design. Importantly, the primary analyses were conducted using trial-level logistic mixed-effects models rather than participant-level regression. Because multilevel models retain within-participant variability and make use of all available trial data without listwise deletion (Quené and van den Bergh, 2004), the regression-based power analysis can be considered a more conservative estimate of the required sample size. Given that we expected some attrition, we recruited more participants than required. Ninety-nine younger adults completed our study, and we eliminated 19 (4 participants failed an attention check, 12 failed to complete Part 2 of the study at the correct delay, and 3 indicated that they had looked up the answers to the trivia questions during the task). Ninety-seven older adults participated, and we eliminated 17 (15 failed to complete Part 2 of the study at the correct delay, 1 indicated that they had looked up the answers to the questions during that task, and 1 provided responses that were unintelligible). This left us with a full sample of 80 younger and 80 older adults recruited from the community via flyers and media postings. Descriptive statistics for sociodemographic measures of samples are presented in Table 1. Pearson correlations between state curiosity, trait curiosity, personality trait, and demographic variables are reported in Table 2. Age was significantly associated with higher Interest- and Deprivation-type trait curiosity as well as education (*ps* < 0.01), indicating that older age was linked to more Interest curiosity and greater educational attainment, but lower Deprivation curiosity. Older age was also associated with higher Openness and Intellect (*ps* < 0.05).

**Table 1:** Sample characteristics and age group differences.

**Variable**	**Younger**	**Older**
	***M (SD)***	***M (SD)***
*N*	80	80
*N* (female)	56	59
Age	20.15 (2.53)	71.29 (7.19)
Age range	18–33	54–94
Education	13.52 (1.49)	16.40 (2.97)
Interest	15.21 (2.95)	16.11 (3.12)
Deprivation	12.74 (3.34)	10.03 (3.34)
Openness	3.75 (0.61)	3.97 (0.47)
Intellect	3.51 (0.61)	3.72 (0.62)

Age and education are measured in years, and interest, deprivation, openness, and intellect are measured as scores (see Materials).

**Table 2:** Correlations between state curiosity, trait curiosity, and demographic variables.

**Variable**	**State curiosity**	**Interest**	**Deprivation**	**Satisfaction**	**Confidence**	**Education**	**Age**	**Intellect**	**Openness**
State curiosity	–								
Interest	0.27^***^	–							
Deprivation	−0.09	0.24^**^	–						
Satisfaction	0.60^***^	0.25^**^	−0.14	–					
Confidence	0.30^***^	0.08	−0.07	0.15	–				
Education	0.27^***^	0.09	−0.12	0.21^**^	0.19^*^	–			
Age	0.42^***^	0.18^*^	−0.36^*^	0.40^***^	0.21^**^	0.53^***^	–		
Intellect	0.17^*^	0.37^***^	0.06	0.22^**^	0.09	0.24^**^	0.18^*^	–	
Openness	0.26^***^	0.43^***^	0.02	0.26^**^	0.01	0.17^*^	0.21^**^	0.43^***^	–

Values are Pearson correlation coefficients.

^*^p < 0.05.

^**^p < 0.01.

^***^p < 0.001.

### Materials

The task consisted of 70 general knowledge questions selected from Kang et al. (2009). For example, “What animal's milk does not curdle?” (Answer: Camel). We also administered a measure of epistemic curiosity (Litman and Silvia, 2006) which includes five items related to Interest-Type epistemic curiosity (motivated by the pleasure of learning something new) and five questions related to Deprivation-Type epistemic curiosity (driven by the desire to resolve a knowledge gap or problem). Participants were asked to rate how often the following statements applied to them (Almost never; Sometimes; Often; Almost always): (1) *I enjoy learning about subjects that are unfamiliar to me;* (2) *I find it fascinating to learn new information;* (3) *I enjoy exploring new ideas;* (4) *When I learn something new, I feel like a whole new world has opened up for me;* (5) *I enjoy discussing abstract concepts;* (6) *I feel frustrated when I can't figure out the solution to a problem;* (7) *I can't rest until I find out the answer to a question that's been bothering me;* (8) *I feel compelled to learn the answer when I encounter a difficult question;* (9) *I work hard to solve problems even when they don't have immediate relevance;*10) *I often find myself thinking about unresolved questions* (1–5 are Interest-Type items). We also administered the Intellect and Openness to Experience subscales of the Big Five Aspect Scale (DeYoung et al., 2007).

### Procedure

The study was conducted online on the Qualtrics platform. Part 1 of the study was the encoding phase: On each trial, participants were presented with a trivia question for 10 s and asked to guess the answer by typing it in. They were then prompted to rate how curious they were to learn the answer on a scale of 1 (*Not at all curious*) to 10 (*Extremely curious*) followed by how confident they were that their guess was correct on a scale of 1 (*Not at all confident*) to 10 (*Extremely confident*). The correct answer to the trivia question was then shown for 6 s after which participants were asked to rate how satisfying this answer was on a scale of 1 (*Not at all satisfying*) to 10 (*Extremely satisfying*). Information prediction error (IPE) was computed by subtracting participants' curiosity ratings from their satisfaction ratings. This was repeated for all 70 trivia questions. Participants were not told that their memory for the answers would be tested. After a delay of 7 days, participants were sent a link for Part 2 of the study. After completing the questionnaires, they did a retrieval task wherein they were shown each trivia question they had seen in Part 1, one at a time, and were asked to recall their associated answers. This task was self-paced, and participants were encouraged to guess if they did not remember. Finally, we asked participants whether they had looked up the answers to the questions during the study so that we could exclude participants who said yes. Participants were compensated $20 for their participation. The study was approved by the Research Ethics Board (certificate #e2018-198).

### Study design

All analyses were conducted using RStudio (Version 2024.04.2.764; R Studio Team, 2020) with a statistical significance threshold set at α = 0.05. Due to the nested structure of trial data within participants, logistic mixed effects models were estimated for the outcome variable (recall), and cluster-mean-centering was applied to the appropriate predictor variables (curiosity, confidence, satisfaction, IPE). In all mixed effects models, random intercepts for the participant (participant ID) and the trivia question (question number) were included (unless variance for either was near-zero). For mixed effects models, the lme4 package (Bates et al., 2015) was used, and fixed effects coefficients were exponentiated to calculate odds ratios (OR). For models containing more than two predictors, no multicollinearity was detected (variance inflation factor <3). Trials wherein participants correctly guessed the trivia question answer were removed in order to examine new learning.

## Results

### Age differences in accuracy, curiosity, satisfaction, and confidence

Pearson correlations between our main variables of interest (trial-level curiosity, satisfaction, confidence, trait curiosity, and personality traits) revealed that older age was associated with significantly higher state curiosity (mean trial-level curiosity reported by participants during the trivia task), satisfaction, and confidence (*ps* < 0.01; Table 2). State curiosity was also significantly associated with both Interest-type trait curiosity and Openness (*ps* < 0.001). For Pearson correlations between curiosity, confidence, and satisfaction variables, see Supplementary Figure S1. A Welch's *t*-test was used to assess age group differences in study accuracy (the proportion of trivia question answers correctly guessed during the study phase). Older adults correctly guessed a significantly greater proportion of trivia questions answers (*M* = 0.12, *SD* = 0.08) during the study phase compared to younger adults (*M* = 0.07, *SD* = 0.07), *t*_(150.71)_ = −3.95, *p* < 0.001, 95% confidence interval (CI) = (−0.07, −0.02).

### Effects of curiosity, confidence, and satisfaction on memory performance

**Figure 1:**
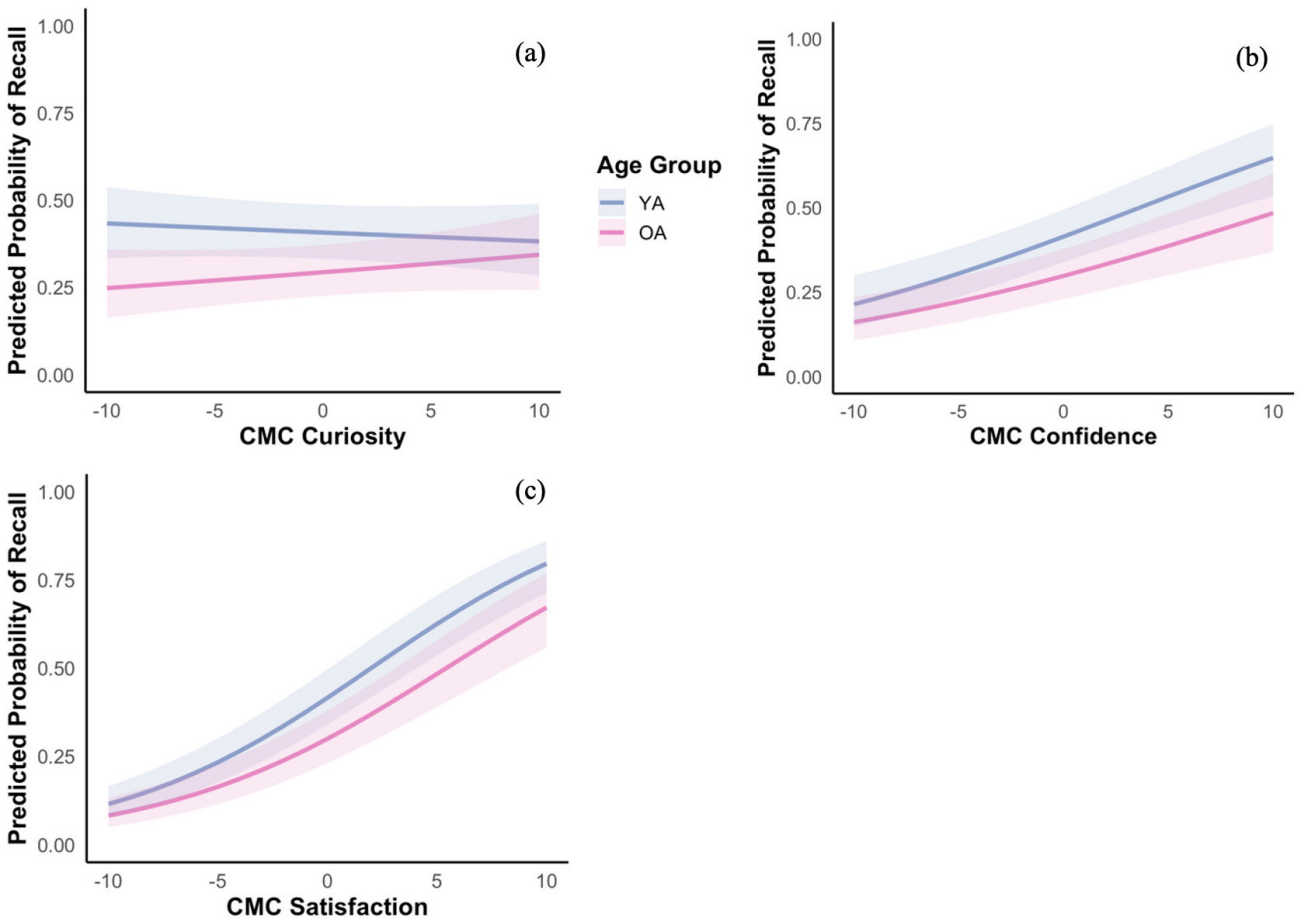
Age differences in predicted probability of recall according to initial curiosity, confidence, and post-answer satisfaction. Age differences in predicted probability of recall are displayed according to cluster-mean-centered (CMC) curiosity **(a)**, confidence **(b)**, and satisfaction **(c)**.

To investigate the effects of curiosity, confidence, and satisfaction on recall memory (the proportion of initially unknown trivia question answers correctly remembered during the recall phase), a mixed effects model was estimated (Figure 1). Recall accuracy was the outcome variable, and cluster-mean-centered curiosity, confidence, and satisfaction were the predictor variables. To determine whether there were age differences in the extent to which curiosity, confidence, and satisfaction predicted recall, age group (younger vs. older adults) was included as an interaction term. Given that age was significantly associated with education (Table 2), we included education as a covariate within the model. We found that age predicted recall performance, β = −0.51, *SE* = 0.17, *z* = −3.04, *p* = 0.002, OR = 0.60, indicating that younger adults displayed higher proportions of recall accuracy than older adults. Education also predicted recall, such that those with higher education displayed enhanced memory for trivia answers, β = 0.11, *SE* = 0.03, *z* = 3.61, *p* < 0.001, OR = 1.12. The results also indicated that while confidence (Figure 1b), β = 0.10, *SE* = 0.02, *z* = 5.68, *p* < 0.001, OR = 1.10, and satisfaction (Figure 1c), β = 0.17, *SE* = 0.02, *z* = 11.22, *p* < 0.001, OR = 1.19, significantly predicted recall, curiosity (Figure 1a) did not, β = −0.01, *SE* = 0.01, *z* = −0.73, *p* = 0.46, OR = 0.99. Importantly, interactions between age group and curiosity, β = 0.03, *SE* = 0.02, *z* = 1.42, *p* = 0.16, OR = 1.03, confidence, β = −0.02, *SE* = 0.02, *z* = −0.70, *p* = 0.48, OR = 0.98, and satisfaction, β = −0.01, *SE* = 0.02, *z* = −0.58, *p* = 0.56, OR = 0.99, were not significant, suggesting that curiosity, confidence, and satisfaction similarly predicted recall memory among younger and older adults.

### Effects of information prediction errors on memory performance

**Figure 2:**
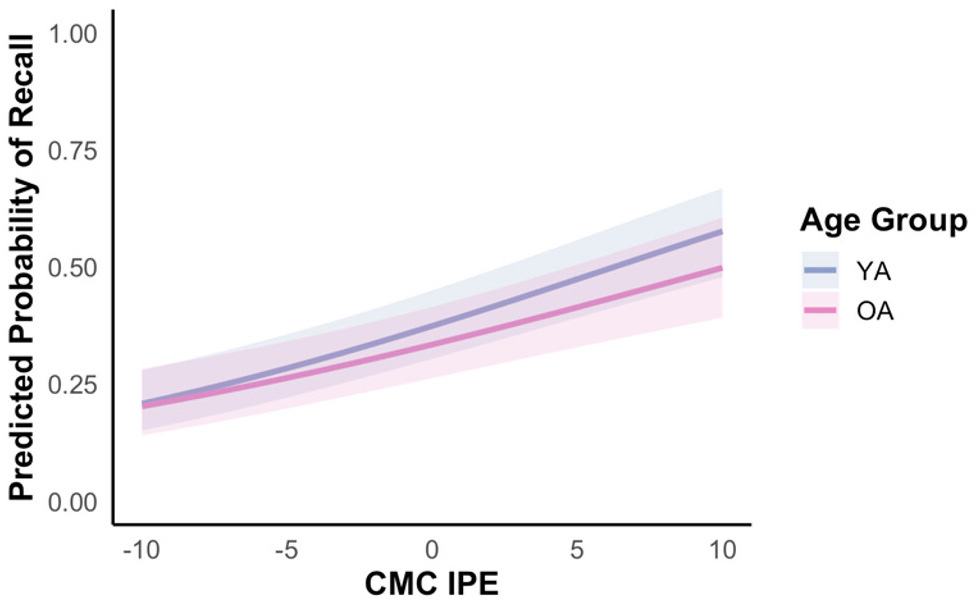
Age differences in predicted probability of recall according to information prediction error (IPE). Age differences in predicted probability of recall are displayed according to cluster-mean-centered (CMC) IPE.

The effects of curiosity and IPE on recall memory were assessed in a model (Figure 2), which included recall as the outcome variable and cluster-mean-centered IPE as the predictor. To determine whether there were age differences in the extent to which IPE predicted recall, age group was included as an interaction term and as in the above model, education was included as a covariate. The results indicated that while IPE, β = 0.08, *SE* = 0.01, *z* = 6.87, *p* < 0.001, OR = 1.09, and education predicted recall, β = 0.12, *SE* = 0.03, *z* = 3.94, *p* < 0.001, OR = 1.13, age did not, β = −0.17, *SE* = 0.17, *z* = −1.05, *p* = 0.30, OR = 84. Critically, the interaction between age and IPE was not significant, β = −0.01, *SE* = 0.02, *z* = −0.76, *p* = 0.45, OR = 0.99, suggesting that IPE similarly predicted recall memory among younger and older adults.

## Discussion

### Aging, satisfaction, state curiosity, and memory

In this well-powered study, we examined age differences in how epistemic emotions like curiosity and satisfaction work independently and jointly to potentiate learning and memory. For both younger and older adults, confidence and post-answer satisfaction in trivia answers predicted recall at 1 week, and the memory-enhancing benefits of both confidence and satisfaction were equivalent between younger and older adults. Notably, curiosity failed to predict recall performance in either age group, although, this may be due to the relationship between curiosity and confidence. For example, a recent study showed that epistemic curiosity is stronger when confidence levels are also high (Sakaki et al., 2024). This finding is consistent with our correlation analyses: There was a strong positive association between state curiosity and confidence ratings, suggesting overlapping variance (Table 2). Accordingly, it is possible that in our model, curiosity did not contribute unique variance to memory performance beyond that which was contributed by confidence Relative to their younger peers, older adults reported higher curiosity, confidence, and satisfaction ratings during the trivia task and had higher baseline knowledge of trivia (0.12 vs. 0.07 of answers guessed correctly). These findings are consistent with McGillivray et al. (2015) and, more broadly, with other work showing no age differences in the relationship between memory and epistemic emotions such as curiosity (Galli et al., 2018; McGillivray et al., 2015; Swirsky et al., 2021; Swirsky and Spaniol, 2024). Together, these results suggest that anticipatory curiosity may not independently drive learning once downstream evaluative processes are taken into account. Specifically, satisfaction emerged as a robust predictor of memory in our model, supporting the idea that post-answer affective evaluations may play a critical role in the consolidation of newly acquired information. These findings align with Fastrich et al. (2018) who conceptualized interest as having both anticipatory (pre-answer) and outcome (post-answer) components. They showed that while pre-answer interest correlates with recall, this relationship is largely mediated by post-answer interest: People tend to remember information better because engaging answers increased their interest after seeing the answer. In their model, however, pre-answer curiosity remained a significant predictor of memory performance though their sample size was significantly larger than ours (*n* = 1,495).

### Aging, IPEs, and memory

We also looked at how the interplay between satisfaction and curiosity may affect learning by calculating so-called information prediction errors. For both age groups, memory was better for trivia answers associated with positive prediction errors, i.e., when satisfaction was higher relative to curiosity. This replicates previous work that has similarly found a positive relationship between IPEs and memory (Fastrich et al., 2018; Marvin and Shohamy, 2016; Van De Cruys et al., 2021) and extends it to older adults. The fact that older adults' memories are just as sensitive to the gap between expected and actual rewards as their younger peers may be surprising at first blush as aging is associated with decreased dopaminergic signaling and changes in feedback processing (Eppinger et al., 2008, 2011). Indeed, Sobczak et al. (2025) found that the surprise caused by trivia answers was less predictive of subsequent memory among older relative to younger adults. However, it is noteworthy that studies have used different terms to assess post-information affect (e.g., interest, satisfaction, surprise) which is used to calculate these so-called prediction errors, and that not all forms of post-answer affect may function identically as learning signals. IPEs are analogous to classical prediction errors in that they reflect a discrepancy between expected and received outcome; however, whereas standard prediction errors are measured at the level of objective rewards or feedback signals, IPEs are subjective and affective. Thus, while IPEs provide a useful framework for linking curiosity and satisfaction to memory, they should not be interpreted as strictly equivalent to standard dopaminergic prediction errors.

Future work would benefit from going beyond behavioral methods to look at how curiosity and satisfaction affect neural substrates of arousal and attention. For instance, Brod and Breitwieser (2019) used pupillometry to show that a learning strategy that led to higher curiosity was accompanied by an increase in pupil dilation both during anticipation of the correct answer and in response to seeing it. Pupillometry may be a good candidate to disentangle how pre- and post-information variables work together to support subsequent memory—especially since pupil dilation is linked to the functioning of the locus coeruleus which undergoes age-related changes (Chen et al., 2025).

Importantly, we also observed converging evidence from the hypercorrection effect in both age groups, i.e., high-confidence (wrong) guesses were more likely to be corrected at recall than low-confidence guesses. This phenomenon has been shown in both younger (Butterfield and Metcalfe, 2001; Fazio and Marsh, 2009) and older adults (Cyr and Anderson, 2013; Eich et al., 2013; Metcalfe et al., 2015) and can be usefully understood as a type of prediction error: A greater mismatch between what one expects to occur and what actually occurs produces a stronger signal to update underlying representations. Our findings on IPEs and hypercorrection converge on the idea that mismatch is a driver of memory updating in both younger and older adults.

### Aging and trait curiosity

We found that older age was positively associated with Interest-Type curiosity (e.g., motivation to learn something new) and negatively associated with Deprivation-Type curiosity (e.g., motivation to find an answer or solution). This former finding contrasts with past studies showing a decrease in trait curiosity among older relative to younger adults (Chu and Fung, 2022; Chu et al., 2020; Robinson et al., 2017; Whatley et al., 2025; Wiegand et al., 2025; Yagi et al., 2023). Future research should investigate whether some curiosity subtypes, such as the exploration of knowledge for pleasure, are more likely to be expressed in older age relative to curiosity that is motivated by a feeling of deprivation (the “itch” to know) which may be felt more aversively. Given motivational shifts with age, we might expect that trait curiosity oriented toward building on prior knowledge and joyful exploration would be more likely to be maintained. For example, recent work (Hirsch et al., 2026; under review) employing the Five-Dimensional Curiosity Scale (Kashdan et al., 2018) found that joyous exploration was significantly higher in older than younger adults, although, deprivation sensitivity was comparable. Finally, we were also interested in whether participants who report higher trait curiosity also show greater curiosity during the trivia task (i.e., state curiosity). We found a significant positive association, replicating Whatley et al. (2025). These results support the view that trait curiosity captures a stable disposition that shapes how individuals engage with specific learning episodes.

An important consideration when interpreting our findings is that our older adult sample appears to be relatively high functioning. This is evidenced by their higher levels of educational attainment and their higher scores on trait Openness/Intellect relative to the younger adult sample. This pattern is noteworthy given prior longitudinal evidence that Openness/Intellect tends to decline after age 65 (Schwaba et al., 2017; Terracciano et al., 2005), suggesting that our older participants may not be representative of older adults more broadly. Higher Openness/Intellect has been linked to greater cognitive reserve and better maintenance of cognitive functioning in later life (Coors et al., 2024), raising the possibility that elevated levels of this trait may have buffered age-related differences in our task. Consistent with this interpretation, a recent study found that education and cognitive reserve among older adults was tied to higher trait curiosity (Wiegand et al., 2025), a construct closely related to Openness/Intellect and intellectual engagement. Indeed, Torenvliet et al. (2025) found that trait Openness was positively associated with curiosity and education. Together, these factors suggest that the relatively high education and curiosity levels of our older adult sample may have contributed to their performance, and partially account for the observed age-related patterns.

### Limitations

The present study has several limitations. First, as is the case with all trivia paradigms, our stimuli included pre-selected general knowledge trivia, which may not arouse the same “need to know” feelings that we encounter in daily instances of curiosity. Future studies should examine the effects of aging on epistemic curiosity using more naturalistic stimuli. For example, Lydon-Staley et al. (2021) had participants freely explore topics in Wikipedia to characterize different patterns of information-seeking that relate to epistemic curiosity. Researching age differences in contexts that similarly enable more open-ended exploration may better inform how curiosity drives learning in everyday environments. Using trivia questions also plays to the strengths of older adults who have richer semantic networks (Rönnlund et al., 2005) and who typically outperform younger adults on measures of general knowledge and vocabulary (Umanath and Marsh, 2012; Salthouse, 2004). Consequently, trivia may be a more engaging set of stimuli for older relative to younger adults. Investigating states of curiosity using non-semantic stimuli (for example, perceptually ambiguous images; e.g., Yagi et al., 2023) may be worth pursuing.

Another limitation is that our samples were not well characterized: We do not report information about ethnicity or income, and we know that samples should reflect the diversity of the studied population. We are aware of one study which found racial differences in state curiosity (Whatley et al., 2025), underscoring the importance of collecting this information. Also, no neuropsychological tests were administered to assess the cognitive status of participants; however, a screening questionnaire confirmed that participants did not have medical or psychiatric conditions known to impact cognition, and the mean total years of education was higher among older adults. As such, we think it unlikely that age differences in our study are due to age-related cognitive deficits among the older adults. Still, future research may explore how the relationship between curiosity and memory may change as a result of cognitive impairment.

## Conclusion

Together, these findings extend prior work by demonstrating age-invariant learning benefits of curiosity and reward satisfaction. Moreover, our results suggest that post-answer satisfaction and positive information prediction errors—reflecting the gap between expected and experienced informational reward—play an important role in learning and memory. Older adults' continued curiosity is nowhere more evident than in the growing popularity of lifelong learning courses, which are now offered at most major universities in Canada. Participants of such programs experience positive effects on their cognitive capabilities (Narushima et al., 2018; Wang et al., 2025) and subjective well-being (Jenkins and Mostafa, 2015; Narushima et al., 2018; Park et al., 2016). As suggested by Sakaki et al. (2018), curiosity may be a proxy for maintaining cognitive functioning and mental health in older adulthood. As such, understanding the role that curiosity plays in aging as well as how to foster it are goals of theoretical and practical importance.

## Data Availability

The datasets presented in this study can be found in online repositories. The names of the repository/repositories and accession number(s) can be found below: https://osf.io/6a7m5/.

## Supplementary material

Supplementary Figure S1 Correlation matrix between curiosity, confidence, and satisfaction. Correlation matrix between cluster-mean-centered (CMC) curiosity, confidence, and satisfaction predictors across age groups.
