# Supplementary figures and images for: Curiosity in younger and older adults: the relationship between information value and memory
Source: Front Cognit. 2026 Mar 13;5:1715793. doi: 10.3389/fcogn.2026.1715793 (PMC13281121; doi:10.3389/fcogn.2026.1715793)

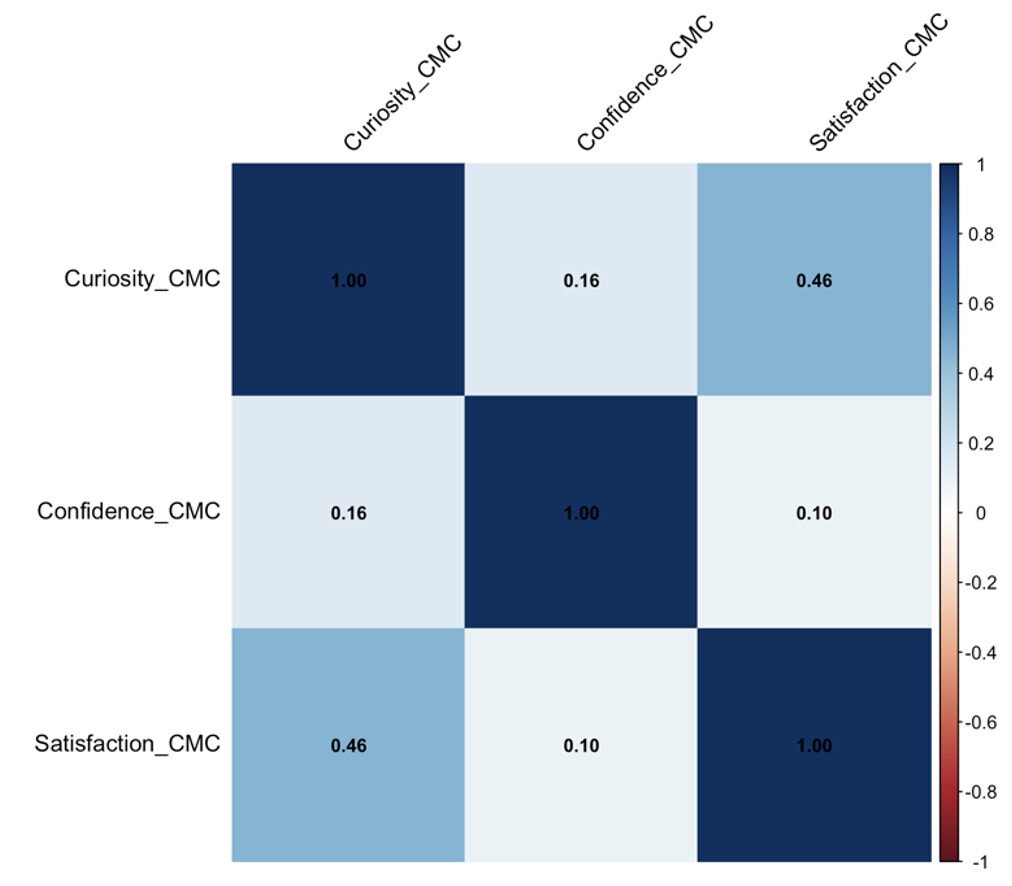

Supplement: Supplementary Figure S1 — Correlation matrix between curiosity, confidence, and satisfaction. Correlation matrix between cluster-mean-centered (CMC) curiosity, confidence, and satisfaction predictors across age groups. [file Image_1.jpg]
